# The association analysis of lncRNA *HOTAIR* genetic variants and gastric cancer risk in a Chinese population

**DOI:** 10.18632/oncotarget.5158

**Published:** 2015-09-04

**Authors:** Mulong Du, Weizhi Wang, Hua Jin, Qiaoyan Wang, Yuqiu Ge, Jiafei Lu, Gaoxiang Ma, Haiyan Chu, Na Tong, Haixia Zhu, Meilin Wang, Fulin Qiang, Zhengdong Zhang

**Affiliations:** ^1^ Department of Environmental Genomics, Jiangsu Key Laboratory of Cancer Biomarkers, Prevention and Treatment, Cancer Center, Nanjing Medical University, Nanjing, China; ^2^ Department of General Surgery, the First Affiliated Hospital of Nanjing Medical University, Nanjing, China; ^3^ Core Laboratory, Nantong Tumor Hospital, Nantong, China; ^4^ Department of Genetic Toxicology, the Key Laboratory of Modern Toxicology of Ministry of Education, School of Public Health, Nanjing Medical University, Nanjing, China

**Keywords:** long noncoding RNA, HOTAIR, genetic variants, gastric cancer

## Abstract

The *HOX transcript antisense intergenic RNA* (*HOTAIR*), a well-known long noncoding RNA, is involved in pathogenesis and progress of multiple tumors. Its ectopic expression and biological functions have been observed in gastric cancer. In this study, we conducted a two-stage case-control study to evaluate whether genetic variations of *HOTAIR* were associated with gastric cancer risk. We identified that a single nucleotide polymorphism (SNP) rs4759314 was significantly associated with the increased gastric cancer risk with an odds ratio (OR) of 1.39 [95% confidence interval (CI) = 1.13–1.71, *P* = 0.002] in the combined sets. Further functional experiments revealed the allele-specific effects on *HOTAIR* and *HOXC11* expressions in gastric cancer tissues, of which *HOTAIR* and *HOXC11* expressions of individuals carrying with AG genotype were much higher than those with AA genotype; similarly, the effects occurred in intronic promoter activities, of which the promoter activity of G allele was more pronounced than that of A allele. Interestingly, we identified a novel potential oncogene *HOXC11* in gastric cancer pathogenesis with differential expression in gastric cancer tissues by association analysis with candidate gene strategy. These results suggest that SNP rs4759314 of *HOTAIR* acts as a potential biomarker for predicting gastric cancer, and the role of *HOXC11* in gastric cancer etiology is warranted to further investigation.

## INTRODUCTION

Gastric cancer (GC) is one of the most common malignant tumors with high mortality in the world. Recently, the GLOBOCAN project (http://globocan.iarc.fr) has reported that the worldwide morbidity of GC is the fifth, and the third for mortality [1]. Although a slight decline in GC incidence and mortality [2], new diagnosed cases and the death each year are still a huge number in Asian countries, predominantly in China, where specifically GC is the third fatal cause, following lung cancer and liver cancer [1]. However, little is known about the exactly mechanism of GC development and progress, despite that accumulating evidences indicate the significant association between environmental factors and epigenetic/genetic effects and GC etiology.

To data, long non-coding RNAs (lncRNAs) have been extremely gained attention for their wide range of biological regulatory functions [3]. Many studies have demonstrated that lncRNAs may be involved in pathogenesis of cancers, referring to the levels of transcription, post-transcription and epigenome [4–6]. Especially, lncRNA *HOX transcript antisense intergenic RNA* (*HOTAIR*) was earlier identified participated in the development and progression of malignancies [4, 7, 8]. Gupta *et al*. initially indicated that *HOTAIR* expression was up-expressed in breast cancer tissues, as well as be related to breast cancer progression [4]; and further studies showed that *HOTAIR* could induce genome-wide retargeting of Polycomb repressive complex 2 (PRC2), contributing to altered H3 lysine 27 methylation [4, 9]. In GC, the *HOTAIR* level was highly expressed in tumors and exerted strong association with clinical phenotypes, such as venous invasion, lymph node metastases and survival [10, 11]; besides, *HOTAIR* could sponge miR-331–3p, functioning as a competing endogenous RNA, to regulate HER2 expression in GC cells [12].

Recently, considerable studies have investigated the effects of lncRNAs genetic variations (majorly composed of single nucleotide polymorphisms, SNPs) on cancer susceptibility. The tagging SNP (tagSNP) rs2839698 of lncRNA *H19* firstly identified was significantly associated with the decreased bladder cancer risk [13], as well that SNP rs6434568 in lncRNA *PCGEM1* was related to prostate cancer risk [14]. In our previous study, we observed a protective effect of SNP rs7958904 in *HOTAIR* on colorectal cancer susceptibility and an allele-specific cellular phenotype in colorectal cancer proliferation [15]. Furthermore, although SNP rs12826786 of *HOTAIR* was related to gastric cardia adenocarcinoma risk, little evidence could support the genetic effects of *HOTAIR* SNPs on GC susceptibility and gene functions. Thus, in this study, we conducted a two-stage case-control study to evaluate the association between *HOTAIR* SNPs and GC risk in a Chinese population, and assess the biological effect of the SNPs.

## RESULTS

### Characteristics of study subjects

In this study, a total of 1275 GC cases and 1646 controls were recruited in two stages, and there were no significant differences between GC cases and cancer-free controls for each stage regarding to age and sex (all *P* > 0.05, Supplementary Table 1). Furthermore, in the combined set, 61.3% of GC cases were in non-cardia type, and 33.6% in the cardia and 5.1% in the both; and a slightly higher percentage was in diffuse type than those in the intestinal (54.4% and 45.6%, respectively). Besides, 23.1%, 24.6%, 35.5%, and 16.8% of GC cases were in TNM stage I, II, III, and IV, respectively.

### Associations of tagSNPs and GC risk

In the test set, the genotypes distributions of 3 SNPs (i.e. rs4759314, rs7958904 and rs874945) among the controls were in accordance with Hardy-Weinberg equilibrium (*P* = 0.699, 0.271 and 0.672, respectively; Supplementary Table 2). Subsequently, four genetic effect models were performed to assess the associations of candidate SNPs and GC risk. We found that only SNP rs4759314 was associated with GC risk (Table 1): although the genotypes frequency distributions of SNP rs4759314 in an additive genetic model was of marginal differences between cases and controls (*P*_trend_ = 0.049), its genetic effect in dominant model significantly associated with GC risk (*P* = 0.030; OR = 1.34, 95% CI = 1.03–1.73), especially the more risk was found in the heterozygotes (OR = 1.36, 95% CI = 1.05–1.77) rather than in the minor homozygotes (OR = 0.75, 95% CI = 0.19–2.99). No significant association with GC risk was observed in both SNPs rs7958904 and rs874945.

**Table 1:** The association between SNP rs4759314 of *HOTAIR* and gastric cancer susceptibility in two-stage case-control study

				MAF^c^	OR (95% CI)^d^			*P*^d^
SNP	Stages	Cases^b^	Controls^b^	Case/Control	Additive/dominant/recessive model	Co-dominant model		Additive
						het^e^	hom^e^	
rs4759314	Test set	624/126/3	915/136/6	0.087/0.070	**1.29 (1.00–1.65)/1.34 (1.03–1.73)**/0.71 (0.18–2.86)	**1.36 (1.05–1.77)**	0.75 (0.19–2.99)	0.047
A/G^a^	Validation set	459/60/3	549/36/2	0.063/0.034	**1.87 (1.26–2.79)/1.99 (1.31–3.04)**/1.73 (0.29–10.38)	**2.01 (1.30–3.09)**	1.84 (0.61–11.05)	0.004
	Combined set	1083/186/6	1464/172/8	0.078/0.057	**1.39 (1.13–1.71)/1.45 (1.17–1.80)**/0.97 (0.34–2.80)	**1.47 (1.18–1.84)**	1.02 (0.35–2.94)	0.002

a A/G represented major allele/minor allele, respectively.

b Genotypes were major homozygote/heterozygote/minor homozygote.

c MAF, minor allele frequency in cancer-free controls.

d OR, odds ratio; CI, confidence interval. *P* was for additive model. The logistic regression analysis was adjusted for age and sex.

e het, heterozygote vs. major homozygote; hom, minor homozygote vs. major homozygote.

Subsequently, we performed an independent validation set and identified that rs4759314 still had significant effects on gastric cancer risk (Table 1). Compared with the AA genotypes, more individuals with G allele were genotyped in cases than that in controls (*P* = 0.004, OR = 1.99, 95% CI = 1.31–3.04 in the dominant model). Similarly, the effect of heterozygotes in co-dominant model was more significant than that of homozygotes (*P* = 0.002 for heterozygote and *P* = 0.506 for homozygote). When combined these two stages, the AG/GG genotypes were also dramatically associated with GC risk (OR = 1.45, 95% CI = 1.17–1.80, *P* < 0.001), and the AG genotype was particular in main effect (OR = 1.47, 95% CI = 1.18–1.84, *P* < 0.001).

**Figure 1:**
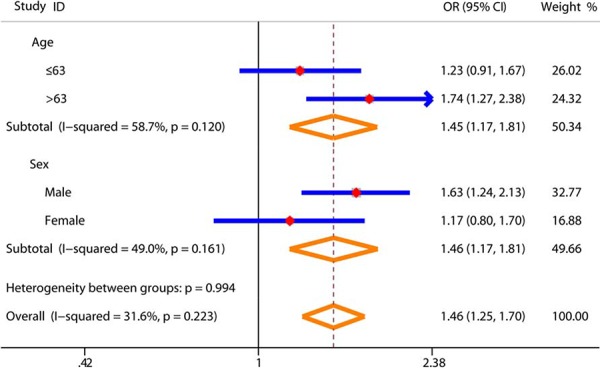
Subgroup analysis of demographic features for the association between SNP rs4759314 and gastric cancer risk in genetic dominant model The red diamonds and blue horizontal lines correspond to the subgroup-specific OR and 95% CI; the orange diamonds represents the pooled OR with 95% CI.

In addition, stratified analyses of SNP rs4759314 were performed by demographic and clinical characteristics. We identified more prominent risk effect of SNP rs4759314 AG/GG genotypes in subgroup of the older subjects (age > 63, *P* < 0.001) and males (*P* < 0.001) (Figure 1), and subsequently found that the AG/GG genotypes were significantly associated with GC risk in each subgroup of tumor site, histological type and TNM stage (Table 2).

**Table 2:** Subgroup analysis of clinical characteristics for the association between SNP rs4759314 and gastric cancer risk

	*n*	OR (95% CI)^a^	AA (*n*, %)	AG/GG (n, %)	*P*^a^
Controls	1646	Reference (1.00)	1464 (89.1)	180 (10.9)	
Tumor site					
Cardia	403	1.58 (1.16–2.15)	339 (84.1)	64 (15.9)	0.006
Non-cardia	734	1.48 (1.15–1.90)	621 (84.6)	113 (15.4)	0.002
Both	61	1.45 (0.70–2.99)	52 (85.3)	9 (14.7)	0.353
Histological type					
Diffuse	612	1.43 (1.09–1.88)	519 (84.8)	93 (15.2)	0.006
Intestinal	513	1.56 (1.17–2.07)	433 (84.4)	80 (15.6)	0.005
TNM stage					
I/II	551	1.51 (1.15–2.00)	465 (84.4)	86 (15.6)	0.004
III/IV	604	1.46 (1.12–1.92)	512 (84.8)	92 (15.2)	0.006

a OR, odds ratio; CI, confidence interval. Adjusted for age and sex in logistic regression model.

### The relationship of SNP rs4759314 on *HOTAIR* and *HOXC11* expression level

**Figure 2:**
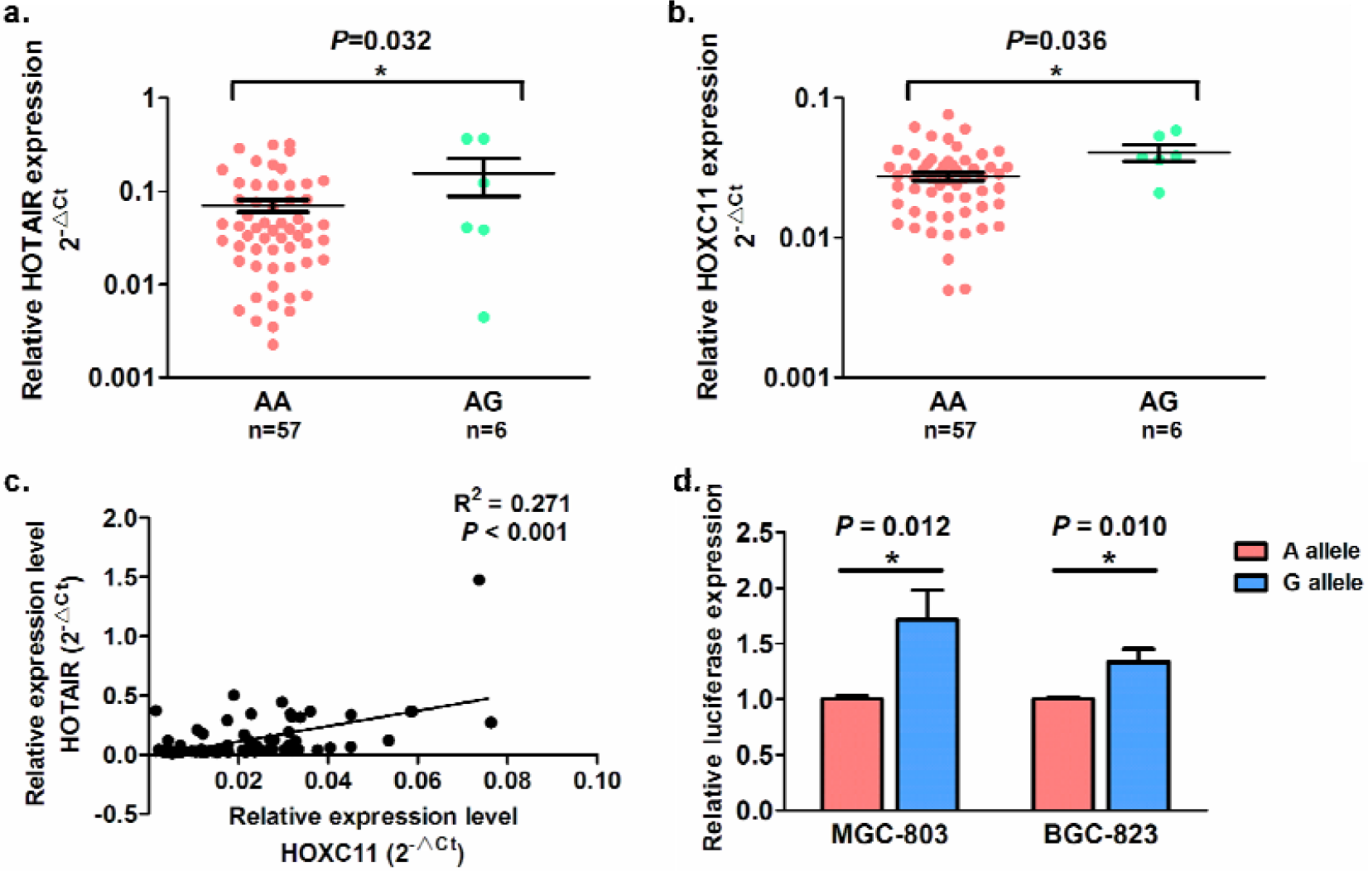
The allele-specific effects of SNP rs4759314 on *HOTAIR* and *HOXC11* expression in 63 gastric cancer tissues and allele-specific promoter activity The allele-specific effects showed significantly on *HOTAIR* **a.** and *HOXC11* **b.** The expression of *HOTAIR* and *HOXC11* presented a slight correlation in gastric cancer tissues **c.** expression level, as well on the intronic promoter activity **d.**

We next investigated whether the GC susceptibility SNP rs4759314 has an allele-specific effect on nearby genes. Supplementary Figure 1 presents the loci of SNP rs4759314 and its nearby genes, thus we measured the expression level of its host gene *HOTAIR* and nearby gene *HOXC11* in 63 GC tissues by using real-time RT-PCR. As shown in Figure 2a–2b, individuals with the AG genotype expressed higher *HOTAIR* and *HOXC11* levels than those with AA genotype in GC tissues (*P* = 0.032 for *HOTAIR* and *P* = 0.036 for *HOXC11*). In addition, we explored The Cancer Genome Atlas (TCGA) database to assess the effect of SNP rs4759314 on *HOXC11* expression level. Similar results were observed that *HOXC11* expression in GC cases with AG genotype was higher than those with AA genotype, although no statistical significance (*P* = 0.144, Supplementary Figure 2a). Furthermore, we evaluated the correlation of these two genes in tumors, and found a positive correlation between *HOTAIR* and *HOXC11* expression level (R^2^ = 0.271, *P* < 0.001, Figure 2c).

### Identification of promoter activity in SNP rs4759314 region

Previous study has reported that *HOTAIR* intron 2 might perform enhancer activity for H3K4me1/3 modification [16]. However, the *HOTAIR* intron 1, GC susceptibility SNP rs4759314 located, did not show any histone modification in this region (Supplementary Figure 1). Intriguingly, we identified that this region might produce promoter activity from ENCODE data; and we therefore examined the promoter activity of intron 1 region by luciferase reporter assays. As shown in Figure 2d, the luciferase activity of intron 1 region containing G allele was significantly higher compared to that of A allele in both MGC-803 and BGC-823 GC cells (*P* = 0.012 and 0.010, respectively), indicating that there might exist pre-transcriptional regulation, especially allele-specific modulation, on *HOXC11* expression in region of *HOTAIR* intron 1, where some allele-specific-binding transcription factors are potentially involved in this region (Supplementary Table 3).

### Candidate genes expression level in GC tissues

Considering several studies have demonstrated abnormal expression of *HOTAIR* in multiple tumors [4, 10, 12, 17], we then evaluated the expression of *HOTAIR*, as well as *HOXC11*, in GC tissues by using Oncomine database, in which datasets from Cho *et al*. [18] and D'Errico *et al*. [19] were selected for further analysis. As shown in Supplementary Figure 3a–3h, both *HOTAIR* and *HOXC11* expression levels in either GC diffuse or intestinal tissues were evidently higher than those in normal gastric tissues. Interestingly, we also observed a slight correlation between the expressions of *HOTAIR* and *HOXC11* (Supplementary Figure 3i–3j). Moreover, the deregulation of *HOXC11* expression was supported by TCGA data, which showed dramatically higher expression of *HOXC11* in GC tissues than that in corresponding adjacent normal tissues (*P* < 0.001, Supplementary Figure 2b).

## DISCUSSION

In the present study, we performed a two-stage case-control study to evaluate the association between three SNPs in *HOTAIR* gene and GC risk. In our findings, SNP rs4759314 A > G was significantly associated with increased GC risk, especially in the elder and male subjects. In addition, the AG genotype was related to higher expression of *HOTAIR* and *HOXC11* in GC tissues, compared to the AA genotype, for which the G allele enhanced higher promoter activity of *HOXC11* than the A allele. This is the first study to assess the genetic role of *HOTAIR* genetic variants in GC susceptibility.

Emerging evidences have demonstrated that *HOTAIR* could act as an oncogene in multiple malignancies and its up-regulated expression may result in malignant transformation of normal cells [8]. Gupta *et al*. firstly identified the abnormal expression of *HOTAIR* in breast cancer tissues, which showed that high expression of *HOTAIR* in breast cancer tissues was associated with poor metastasis-free survival and overall survival [4]. Subsequently, similar phenomena have been identified in other solid tumors. For GC, the *HOTAIR* level was highly expressed and significantly associated with gastric cancer progresses and prognosis [10, 12, 20, 21]. In this study, we identified the association of rs4759314 G allele and increased *HOTAIR* expression in GC tissues, which indicated biological rationality of this correlation.

Recently, several studies have investigated the genetic effects of *HOATIR* tagSNPs on cancer susceptibility. Zhang *et al*. initially identified 3 tagSNPs of *HOTAIR* gene (i.e., rs920778, rs1899663 and rs4759314) and evaluated their associations on esophageal squamous cell carcinoma (ESCC) risk; and they found that SNP rs920778 was dramatically related to increased ESCC risk and functional study demonstrated an allele-specific effect on the intronic enhancer activity of *HOTAIR* [16]. Subsequently, Guo *et al*. assessed the relationship between *HOTAIR* polymorphisms and gastric cardia adenocarcinoma (GCA) risk, and identified that tagSNP rs12826786 not only was associated with increased GCA risk, but also performed a genotype-specific effect on *HOTAIR* expression [22]. In present study, although two of three tagSNPs were not the same as reported tagSNPs, they were in the same block region [15] and showed high correlation (1.0 of r^2^ between rs7958904 and rs920778; 1.0 for rs874945 and rs12826786; Supplementary Table 4). Given these, our identified risk SNP rs4759314 in GC susceptibility was quite different from previously reported risk SNPs. Additionally, in our previous study, we observed a significant association between the SNP rs7958904 and decreased risk of colorectal cancer and found an allele-specific phenotype of SNP rs7958904 in colorectal cancer cell proliferation [15]. All of these indicated an important role of *HOTAIR* genetic variants in cancer susceptibility.

Considering that recognizing the precise targets of risk variants located in noncoding regions was still the major challenge [23], we hypothesized that the risk SNP rs4759314 falling in intron of *HOTAIR* gene had a genotype-phenotype correlation on its nearby genes. Intriguingly, we observed that the intron region of SNP rs4759314 resided performed a promoter activity from ENCODE data, and gene *HOXC11* was only located in about 5-kb downstream of SNP rs4759314, which was involved in the development and outcome of cancer [24]. We hypothesized whether the intronic region of SNP rs4759314 resided existed allele-specific mediated promoter activity [25]. Thus, we explored the potential transcription factors on this candidate intron of *HOTAIR* by two online bioinformatics tools, from which some conventional transcription factors were predicted and also were affected by SNP rs4759314 alleles. This phenotype was validated in further functional experiments: the luciferase activity of risk allele G of rs4759314 was higher than that of A allele, and SNP rs4759314 presented an genotype-specific expression on *HOXC11* expression. Besides, this phenomenon was supported by TCGA database, although no statistical significance in genotype-specific expression analysis, which might be for genetic ancestry of European populations (minor allele frequency = 0.017 of SNP rs4759314 G allele from CEU populations of 1000 Genomics Project) is different from that of Asians (0.100 of G allele in CHB and JPT populations).

In conclusion, we identified that a functional SNP rs4759314 of *HOTAIR* had a strong association with GC susceptibility in the Chinese populations. SNP rs4759314 resided in an intronic promoter region influenced the activity of this promoter, contributing to a genotype-specific effect on the expression of host gene *HOTAIR* and its downstream gene *HOXC11*, which is a potential mechanism for GC susceptibility. All these indicated that *HOTAIR* and its genetic variations may be a potential biomarker for risk assessment, early detection and therapeutic target of gastric cancer; and larger prospective and experimental studies were warranted to further validation.

## MATERIALS AND METHODS

### Study subjects

This study was approved by the Institutional Review Board of Nanjing Medical University. All subjects enrolled were heritably unrelated ethnic Han Chinese. The patients were recruited in our ongoing GC study, in which individuals were from the Second Affiliated Hospital of Nanjing Medical University, Cancer Hospital of Nantong and Yixing Cancer Hospital from March 2006 to January 2011 which described in detail previously [26, 27]. Briefly, a total of 1,275 GC cases and 1,646 cancer-free controls were recruited in two stages (753 cases and 1,057 controls for the test set, with additional 522 cases and 589 controls for the validation set). The controls frequency-matched to cases on age (±5 years) and sex were randomly enrolled in the same hospital for a routine examination of physical conditions. All the patients were histologically confirmed and the clinical information was obtained, including tumor site (cardia/non-cardia/both), histological type and clinical tumor node metastasis (by UICC/AJCC criteria of TNM stage).

### SNP selection and genotyping

In our previous study, we have identified three tagSNPs of *HOTAIR* gene (i.e., rs4759314, rs7958904 and rs874945) [15]. All samples were genotyped by TaqMan allelic discrimination methods with ABI 7900HT real-time PCR system (Applied Biosystems, Foster City, CA, USA). The average call rates for each SNP were over 98%; the random 10% of samples were repeatedly genotyped and the concordance rate was 100%.

### Analysis of *HOTAIR* and *HOXC11* expression in gastric cancer tissues

A total of 63 GC tissues were obtained from pre-treatment patients. Total RNA were extracted from tissues by using TRIzol reagent (Invitrogen, Carlsbad, CA, USA) according to the protocol. The cDNA was then synthesized with M-MLV reverse transcriptase (Invitrogen). Subsequently, the expression of *HOTAIR* and *HOXC11* were determined by SYBR Green Assay (TaKaRa Biotechnology, Dalian, China) and the levels were normalized by Glyceraldehyde 3-phosphate dehydrogenase (GAPDH) by the 2^−ΔCt^ method. All assays were conducted by using the ABI 7900 HT real-time PCR system (Applied Biosystems, Foster City, CA, USA). All reactions were performed in triplicate.

### Detecting promoter activity of SNP rs4759314

The luciferase reporter assay was used to detect the effect of SNP rs4759314 on *HOTAIR* promoter activity. The sequences of target intron with SNP rs4759314 A or G alleles were gene-synthesized and then were cloned into Xho l/Hind III restrictive sites of pGL3-enhancer plasmid (Promega, Madison, WI, USA), and the accuracy was confirmed by DNA sequencing. MGC-803 and BGC-823 cells were cultured in 24-well plates and transfected with 800 ng of each allele-specific plasmids using Lipofectamine™ 2000 (Invitrogen, Carlsbad, CA, USA), respectively. The pRL-SV40 (Promega, Madison, WI, USA) as internal control was cotransfected into cells. After 48 h transfection, dual luciferase activities were determined using a Luciferase Reporter Assay System (Promega, Madison, WI, USA). All assays were conducted in independent triplicate.

### Statistical analysis

Differences in the distribution of selected demographic variables and genotypes of tagSNPs were evaluated by Pearson's χ^2^ test. Hardy-Weinberg equilibrium (HWE) for each SNP among controls was tested using a goodness-of-fit χ^2^-test. The associations of each SNP and GC susceptibility were estimated by using unconditional logistic regression analyses with odds ratios (ORs) and 95% confidence intervals (CIs). Variables of age and sex were as covariates adjusted for the association analysis. Multiple genetic models (i.e., additive, dominant, recessive and co-dominant models) were applied to assess the significance of SNPs. In additional subgroup analysis, the heterogeneity was evaluated by the χ^2^ based on *Q*-test. Differences in allele-specific promoter activity and gene expression were compared by Student's *t*-test or paired *t*-test. A *P* value of less 0.05 for two-side was considered statistically significant. All analyses were conducted with SAS 9.1.3 software (SAS Institute, Inc., Cary, NC, USA).

## SUPPLEMENTARY FIGURES AND TABLES


