# Supplementary material for: The association analysis of lncRNA HOTAIR genetic variants and gastric cancer risk in a Chinese population
Source: Oncotarget. 2015 Sep 4;6(31):31255–62. doi: 10.18632/oncotarget.5158 (PMC4741602; doi:10.18632/oncotarget.5158)
Supplement: Supplementary file 1 [file oncotarget-06-31255-s001.pdf]

SUPPLEMENTARY TABLES AND FIGURES

Supplementary Table S1: Demographic characteristics and clinical features of test and validation sets among gastric cancer cases and caner-free controls.

Variables	Test set			Validation set			Combined set		
	Cases	Controls		Cases	Controls		Cases	Controls	
	n = 753 (%)	*n* = 1057 (%)	*P*[a]	*n* = 522 (%)	*n* = 589 (%)	*P*[a]	*n* = 1275 (%)	*n* = 1646 (%)	*P*[a]
Age (years)	62.9 ± 10.4	62.0 ± 13.0		63.4 ± 11.1	60.8 ± 15.0		63.1 ± 10.7	61.6 ± 13.8	
≤63	381 (50.6)	522 (49.4)	0.611	257 (49.2)	276 (46.9)	0.429	638 (50.0)	798 (48.5)	0.404
>63	372 (49.4)	535 (50.6)		265 (50.8)	313 (53.1)		637 (50.0)	848 (51.5)	
Sex									
Male	512 (68.0)	680 (64.3)	0.105	367 (70.9)	430 (73.0)	0.425	879 (69.2)	1110 (67.4)	0.322
Female	241 (32.0)	377 (35.7)		151 (29.1)	159 (27.0)		392 (30.8)	536 (32.6)	
NA				4			4		
Tumor site									
Cardia	295 (39.2)			109 (24.3)			403 (33.6)		
Non-cardia	432 (57.4)			302 (67.4)			734 (61.3)		
Both	26 (3.4)			37 (8.3)			61 (5.1)		
NA				74			74		
Histological type									
Diffuse	409 (57.8)			203 (48.7)			612 (54.4)		
Intestinal	299 (42.2)			214 (51.3)			513 (45.6)		
NA	45			105			150		
TNM stage									
I	190 (26.8)			77 (17.3)			267 (23.1)		
II	155 (21.9)			129 (28.9)			284 (24.6)		
III	251 (35.4)			159 (35.7)			410 (35.5)		
IV	113 (15.9)			81 (18.1)			194 (16.8)		
NA	44			76			120		

[a]*P* for two-sided x^2 test.

Supplementary Table S2: The association between *HOTAIR* genetic variations and gastric cancer risk in the test set.

tagSNPs (Major/ Minor)	Location[a]	Position	Cases $n = 753$ AA/AB/ BB	Controls $n = 1057$ AA/AB/ BB	P(HWE)	MAF Case/ Control	MAF 1000 Genome Project[b] CHB/CEU/ YRI	P^c Additive/ Dominant/ Recessive model	P_{trend}^c	OR (95% CI)[d] Additive/ dominant/ recessive model	OR (95% CI)[d] Co-dominant model AB vs. AA	OR (95% CI)[d] Co-dominant model BB vs. AA
rs4759314 (A > G)	chr12: 54,361,835	intron	624/126/3	915/136/6	0.699	0.087/ 0.070	0.100/0.017/ 0.322	0.064/**0.030**/ 0.614	**0.049**	**1.29 (1.00–1.65)/ 1.34 (1.03–1.73)/** 0.71 (0.18–2.86)	**1.36 (1.05–1.77)**	0.75 (0.19–2.99)
rs7958904 (G > C)	chr12: 54,357,552	exon6	412/276/51	568/404/85	0.271	0.256/ 0.272	0.217/0.292/ 0.932	0.561/0.399/ 0.369	0.299	0.92 (0.80–1.07)/ 0.92 (0.76–1.11)/ 0.85 (0.59–1.22)	0.91 (0.74–1.11)	0.80 (0.56–1.16)
rs874945 (G > A)	chr12: 54,355,451	3′-flanking region	495/225/31	714/307/36	0.672	0.191/ 0.179	0.117/0.267/ 0.449	0.632/0.466/ 0.423	0.372	1.08 (0.91–1.28)/ 1.08 (0.88–1.31)/ 1.22 (0.75–1.99)	1.05 (0.85–1.29)	1.24 (0.76–2.03)

AA, major homozygote; AB, heterozygote; BB, minor homozygote.
HWE, Hardy-Weinberg equilibrium in controls.
MAF, minor allele frequency in controls.
OR, odds ratio; CI, confidence interval.
[a]Location in GRCh 37.
[b]Allele frequency corresponds to our study.
[c]P for two-sided x^2 test.
[d]Adjusted for age and sex in logistic regression model.

Supplementary Table S3: The prediction of transcription factors on SNP rs4759314 region.

rs4759314	AliBaba 2.1	Alggen
G allele	Oct-1; c-Myc; Sp1	PR_B; PR_A; ENKKTF-1; R2; Sp1; E47; AP-2αA
A allele	Oct-1; Sp1	PR_B; PR_A; ENKKTF-1; NFI/CTF; AP-2αA; NF-1

AliBaba 2.1: http://www.gene-regulation.com/pub/programs/alibaba2/index.html
alggen: http://alggen.lsi.upc.es/

Supplementary Table S4: The correlation of previous reported SNPs in *HOTAIR* and the SNPs in this study.

Current SNPs	Zhang *et. al.* 2014			Guo *et. al.* 2014		
	rs4759314	rs920778	rs1899663	rs10783618	rs4759314	rs12826786
rs4759314	—	—	—	—	—	—
rs7958904	—	*r2* = 1.0	—	—	—	—
rs874945	—	—	*r2*=0.904	—	—	*r2*=1.0

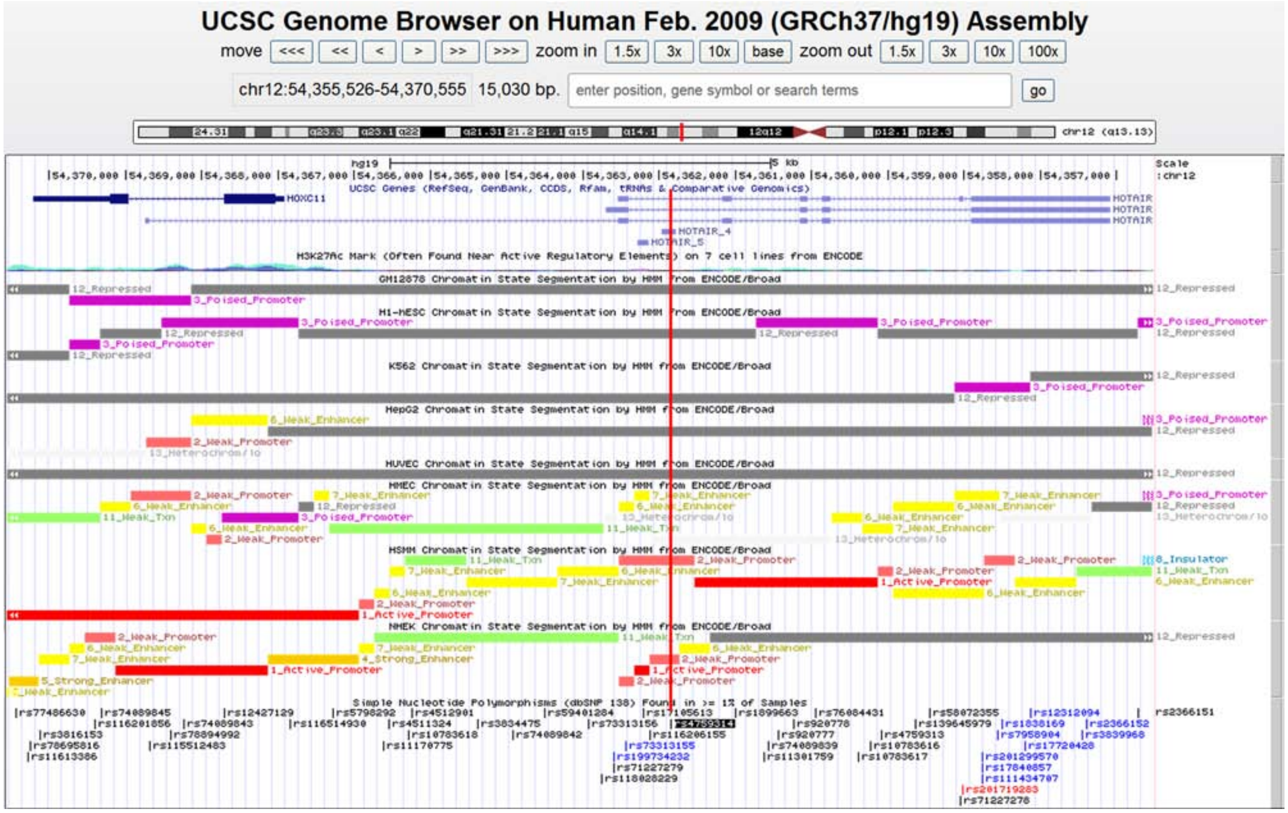

Supplementary Figure S1: The functional annotation of SNP rs4759314 in UCSC Genome Browser. The red line represented the location of SNP rs4759314 in the genome. SNP rs4759314 was located in the intron of *HOTAIR* gene, just the potential promoter region of *HOXC11* resided.

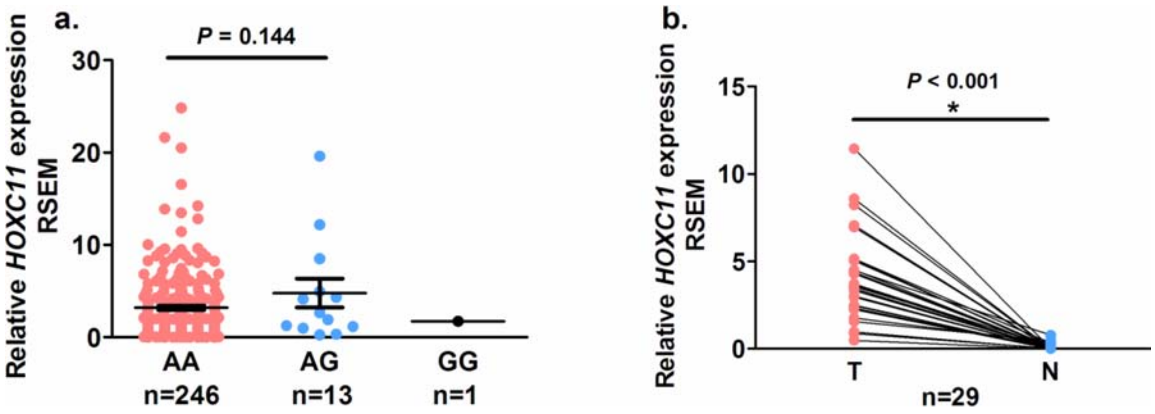

Supplementary Figure S2: The genetic effect of SNP rs4759314 on *HOXC11* expression in The Cancer Genome Atlas database. **a.** for allele-specific effect on *HOXC11* expression in gastric cancer tissues; **b.** for the differential expression of *HOXC11* in gastric cancer tissues and corresponding normal tissues. RSEM, RNA-Seq by Expectation-Maximization; T, tumor tissues; N, normal tissues.

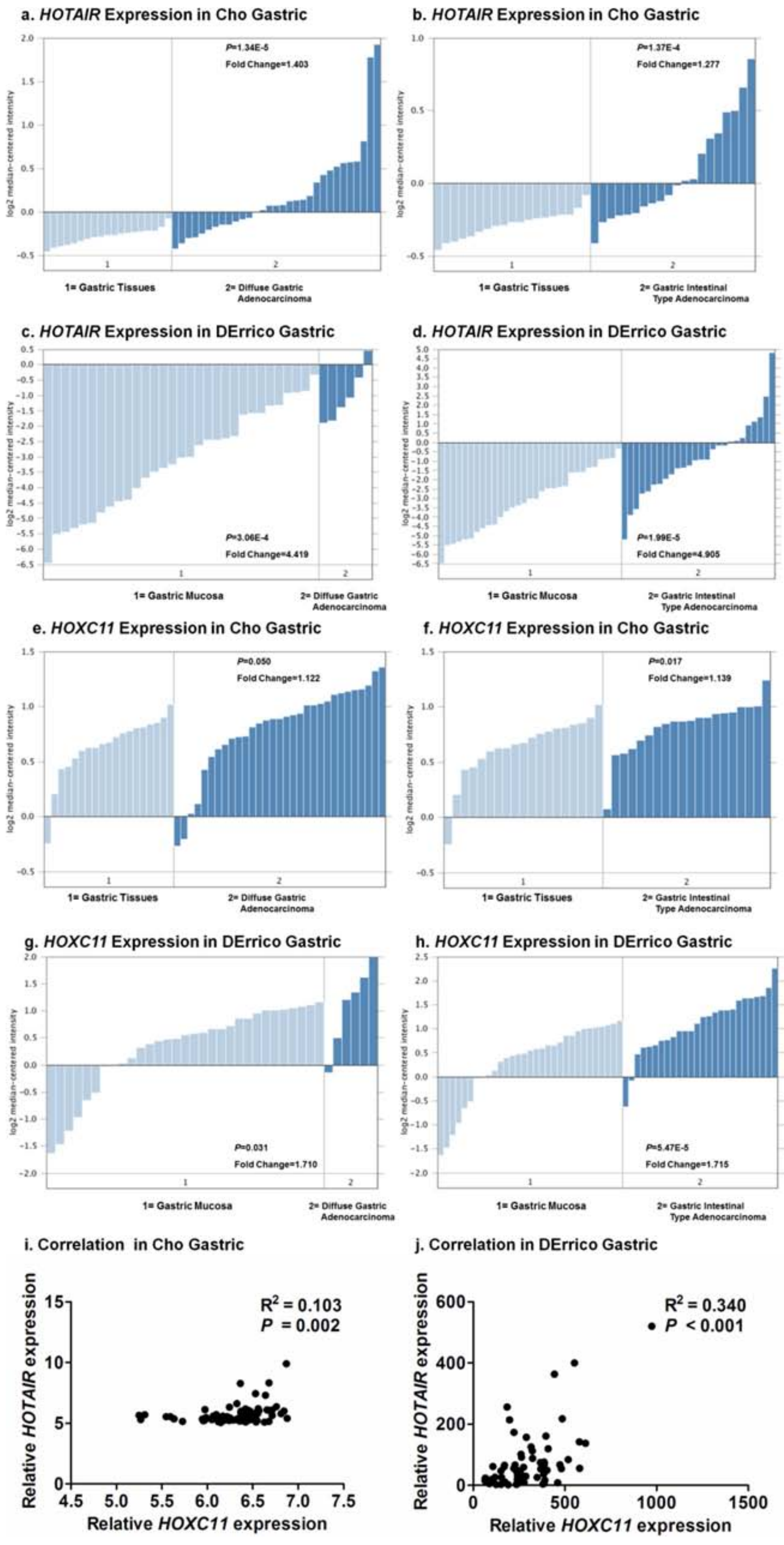

Supplementary Figure S3: The expression levels of *HOTAIR* and *HOXC11* based on Oncomine database. a–d. for *HOTAIR* expression; **e–h.** for *HOXC11* expression; **i–j.** for the correlation of *HOTAIR* and *HOXC11* in the each dataset.
